# Supplementary material for: Association between chronic obstructive pulmonary disease (COPD) and occupational exposures: A hospital based quantitative cross-sectional study among the Bangladeshi population
Source: PLoS One. 2020 Sep 23;15(9):e0239602. doi: 10.1371/journal.pone.0239602 (PMC7510960; doi:10.1371/journal.pone.0239602)
Supplement: S1 Table — (PDF) [file pone.0239602.s001.pdf]

S1 Table: Spirometry values of control participants according to self-reported occupational exposure.

	Exposed										Unexposed									
	Pre-BD					Post-BD					Pre-BD					Post-BD				
No.	FEV1	FVC	FEV1/FVC	%FEV1 predicted	%FVC predicted	FEV1	FVC	FEV1/FVC	%FEV1 predicted	% FVC predicted	FEV1	FVC	FEV1/FVC	%FEV1 Predicted	% FVC Predicted	FEV1	FVC	FEV1/FVC	%FEV1 Predicted	% FVC predicted
01	2.88	3.95	72.91	77.85	85.98	3.1	4	77.50	83.80	87.07	3.5	3.88	90.21	98.30	87.85	3.81	4.01	95.01	107.01	90.79
02	3.7	3.87	95.61	99.83	84.97	3.71	4.01	92.52	100.10	88.05	3.55	3.8	93.42	126.33	103.78	3.6	3.9	92.31	128.11	106.51
03	2.88	3.46	83.24	87.73	85.17	2.9	3.5	82.86	88.34	86.16	3.45	4.4	78.41	127.32	129.21	3.8	4.6	82.61	140.24	135.08
04	3.44	4.36	78.90	109.65	111.09	3.5	4.4	79.55	111.56	112.11	3.65	4.33	84.30	135.17	123.29	3.7	4.4	84.09	137.02	125.28
05	3.02	3.02	100.00	100.95	79.74	3.07	3	102.33	102.62	79.21	3.4	4.24	80.19	123.56	117.56	3.5	4.2	83.33	127.19	116.45
06	2.88	3.01	95.68	113.03	88.17	3.01	3.03	99.34	118.14	88.75	3.15	4.45	70.79	119.22	128.72	3.2	4.5	71.11	121.12	130.17
07	2.76	4.12	66.99	90.50	107.23	2.9	4.1	70.73	95.09	106.71	3.65	4.55	80.22	134.70	133.61	3.8	4.6	82.61	140.24	135.08
08	3.65	3.07	118.89	115.45	78.60	3.76	3.08	122.08	118.93	78.86	3.67	4.78	76.78	116.08	122.38	3.9	4.9	79.59	123.35	125.45
09	2.87	4.12	69.66	105.92	120.99	3	4.1	73.17	110.72	120.40	2.9	2.11	137.44	120.38	65.19	3.2	2.5	128.00	132.83	77.24
10	2.89	4.05	71.36	106.66	118.93	3	4.1	73.17	110.72	120.40	3.4	4.35	78.16	103.57	107.08	3.5	4.4	79.55	106.61	108.31
11	3.67	4.28	85.75	116.08	109.58	3.7	4.3	86.05	117.03	110.09	3.02	2.65	113.96	111.45	77.82	3.06	2.7	113.33	112.93	79.29
12	2.89	3.78	76.46	110.60	110.22	3	3.8	78.95	114.81	110.80	2.98	3.9	76.41	114.95	109.91	3.1	4	77.50	119.57	112.73
13	2.89	3.98	72.61	84.29	94.76	3	4.01	74.81	87.50	95.48	3	4.55	65.93	110.72	133.61	3.25	4.3	75.58	119.94	126.27
14	2.9	4.25	68.24	107.03	124.81	3.1	4.3	72.09	114.41	126.27	3.87	4.65	83.23	110.50	106.61	4	4.7	85.11	114.21	107.76
15	2.98	4.25	70.12	109.98	124.81	3.1	4.3	72.09	114.41	126.27	2.95	3.05	96.72	75.58	63.72	3.18	3.02	105.30	81.47	63.09
16	3.87	4.4	87.95	122.41	112.65	4	4.5	88.89	126.52	115.21	3.45	4.25	81.18	116.72	111.86	3.6	4.3	83.72	121.80	113.18
17	3.76	4.15	90.60	132.62	117.09	3.8	4.2	90.48	134.03	118.50	3.77	4.3	87.67	106.55	98.86	3.9	4.4	88.64	110.22	101.16
18	3.01	3.76	80.05	131.91	129.42	3.18	3.8	83.68	139.37	130.80	3.76	4.5	83.56	138.76	132.15	4.1	4.5	91.11	151.31	132.15
19	3.68	4.15	88.67	135.81	121.87	3.7	4.2	88.10	136.55	123.34	4.12	4.65	88.60	117.18	108.58	4.5	4.8	93.75	127.99	112.08
20	2.56	3.56	71.91	90.29	100.44	2.6	3.6	72.22	91.70	101.57	4.01	4.8	83.54	115.97	113.54	4.2	4.9	85.71	121.47	115.91
21	3.01	3.49	86.25	95.06	88.30	3.07	3.51	87.46	96.96	88.81	3.67	4.35	84.37	122.68	114.86	3.7	4.4	84.09	123.68	116.18
22	3.44	4.15	82.89	108.80	106.25	3.5	4.3	81.40	110.70	110.09	3.88	4.35	89.20	119.50	106.76	3.9	4.5	86.67	120.11	110.44
23	2.98	3.75	79.47	90.77	92.31	3.1	3.7	83.78	94.43	91.08	3.29	4	82.25	90.19	88.90	3.41	3.98	85.68	93.48	88.46
24	2.78	3.86	72.02	91.36	99.44	2.98	3.91	76.21	97.93	100.73	2.98	4.15	71.81	84.22	95.41	3.1	4.2	73.81	87.61	96.56
25	2.76	3.71	74.39	98.22	101.32	2.81	3.78	74.34	100.00	103.23	3.54	4.45	79.55	114.14	113.03	3.7	4.5	82.22	119.30	114.30
26	2.78	3.14	88.54	103.02	94.40	2.98	3.2	93.13	110.43	96.21	3.45	4.45	77.53	143.21	137.48	3.75	4.5	83.33	155.66	139.02
27	3.04	3.75	81.07	112.19	110.12	3.21	3.78	84.92	118.47	111.00	3.46	4.34	79.72	101.99	103.04	3.6	4.4	81.82	106.11	104.46
28	3.45	4.05	85.19	127.32	118.93	3.5	4.1	85.37	129.17	120.40	3.77	4.65	81.08	132.97	131.20	3.9	4.7	82.98	137.56	132.61
29	3.11	4.1	75.85	92.46	97.98	3.2	4.2	76.19	95.14	100.37	3	3.7	81.08	109.02	102.59	3.1	3.7	83.78	112.65	102.59
30	3.01	3.93	76.59	84.22	90.60	3.07	4.01	76.56	85.89	92.45	3.7	4.55	81.32	104.37	105.57	3.9	4.7	82.98	110.01	109.05
31	2.78	3.93	70.74	98.93	107.33	3	4.01	74.81	106.76	109.51	3.84	3.98	96.48	141.72	116.88	3.89	4.01	97.01	143.56	117.76
32	2.89	3.8	76.05	99.57	107.99	3.03	4.01	75.56	104.40	113.96	3.82	4.6	83.04	120.82	117.77	4.1	4.7	87.23	129.68	120.33
33	3.1	3.88	79.90	99.96	98.56	3.2	4	80.00	103.18	101.60	3.76	4.55	82.64	107.15	105.27	3.9	4.7	82.98	111.14	108.74
34	2.89	3.81	75.85	91.41	97.55	3	3.91	76.73	94.89	100.11	2.78	3.8	73.16	102.60	111.59	2.91	3.84	75.78	107.39	112.77
35	3	4.21	71.26	93.48	107.93	3.1	4.2	73.81	96.60	107.67	3.44	4.25	80.94	106.90	105.02	3.6	4.3	83.72	111.88	106.25
36	2.67	2.69	99.26	98.54	78.99	2.81	2.67	105.24	103.70	78.41	2.65	3.9	67.95	75.52	90.23	2.86	3.82	74.87	81.50	88.38
37	3.78	4.35	86.90	111.42	103.27	3.8	4.4	86.36	112.01	104.46	3.67	4.45	82.47	154.65	136.97	3.9	4.5	86.67	164.34	138.51
38	2.78	3.65	76.16	81.08	86.90	2.9	3.7	78.38	84.58	88.10	3.76	4.5	83.56	138.76	132.15	3.9	4.6	84.78	143.93	135.08
39	3.78	3.96	95.45	126.43	109.96	3.81	4.01	95.01	127.44	111.35	3.87	4.55	85.05	142.82	133.61	4.1	4.6	89.13	151.31	135.08
40	2.78	3.78	73.54	92.93	99.81	2.98	3.81	78.22	99.61	100.60	2.34	2.82	82.98	79.82	79.52	2.39	2.8	85.36	81.53	78.96
41	3.71	4.33	85.68	110.59	107.22	3.8	4.4	86.36	113.27	108.96	2.89	3.78	76.46	102.85	103.23	3.1	3.9	79.49	110.32	106.51
42	3.44	3.96	86.87	126.95	116.29	3.46	4.01	86.28	127.69	117.76	3.65	4.35	83.91	134.70	127.74	3.8	4.4	86.36	140.24	129.21
43	3.11	4.35	71.49	103.96	114.86	3.2	4.4	72.73	106.97	116.18	3.88	4.55	85.27	122.72	116.49	4	4.6	86.96	126.52	117.77
44	2.89	4.15	69.64	106.66	121.87	3.1	4.2	73.81	114.41	123.34	3.78	4.35	86.90	119.57	111.63	4.1	4.5	91.11	129.69	115.48
45	2.67	3.66	72.95	91.23	97.04	2.98	3.71	80.32	101.83	98.36	3.12	3.84	81.25	115.14	112.77	3.21	3.91	82.10	118.47	114.82
46	2.55	3.35	76.12	75.16	79.53	2.82	3.3	85.45	83.12	78.35	3.04	4.12	73.79	93.63	101.12	3.2	4.1	78.05	98.55	100.63
47	2.77	3.22	86.02	96.87	90.19	2.98	3.27	91.13	104.21	91.59	3.78	4.32	87.50	139.50	126.86	3.9	4.4	88.64	143.93	129.21
48	2.67	3.62	73.76	95.84	106.20	2.8	3.7	75.68	100.51	108.55	3.1	3.77	82.23	105.19	103.20	3.2	3.8	84.21	108.59	104.02
49	3.04	4.15	73.25	99.68	108.01	3.1	4.2	73.81	101.64	109.31	3.67	4.25	86.35	116.98	108.29	3.9	4.3	90.70	124.31	109.56
50	3.1	4.45	69.66	114.41	130.68	3.3	4.5	73.33	121.79	132.15	3.65	4.1	89.02	134.70	120.40	3.7	4.2	88.10	136.55	123.34
51	3.56	4.45	80.00	128.33	121.13	3.8	4.5	84.44	136.98	122.49	2.78	3.61	77.01	97.68	98.91	2.98	3.71	80.32	104.71	101.65
52	2.88	3.75	76.80	91.80	95.55	3	3.8	78.95	95.62	96.82	3.76	4.1	91.71	103.07	91.12	3.9	4.2	92.86	106.91	93.35
53	2.67	3.14	85.03	90.33	82.65	2.72	3.28	82.93	92.03	86.33	3.81	3.96	96.21	117.34	97.19	3.89	4.01	97.01	119.80	98.42
54	3.01	3.8	79.21	111.08	111.59	3.08	3.87	79.59	113.67	113.65	4.01	4.65	86.24	147.99	136.55	4.2	4.7	89.36	155.00	138.02
55	3.12	4.25	73.41	115.14	124.81	3.2	4.3	74.42	118.10	126.27	3.89	4.45	87.42	123.04	113.93	4.2	4.6	91.30	132.84	117.77
56	2.55	3.52	72.44	94.11	103.37	2.6	3.5	74.29	95.95	102.78	3.67	4.1	89.51	133.37	113.68	3.7	4.3	86.05	134.46	119.22
57	3.5	4.45	78.65	97.92	102.59	3.6	4.5	80.00	100.72	103.74	3.12	3.73	83.65	96.09	91.55	3.21	3.78	84.92	98.86	92.77
58	3.3	3.96	83.33	97.08	94.91	3.35	4.01	83.54	98.55	96.11	2.87	3.68	77.99	107.44	105.61	2.91	3.71	78.44	108.94	106.47
59	3.98	3.76	105.85	123.95	92.01	4.01	3.81	105.25	124.88	93.23	3.15	3.76	83.78	97.01	92.28	3.21	3.78	84.92	98.86	92.77
60	3.56	4.15	85.78	131.83	118.17	3.6	4.2	85.71	133.32	119.59	3.76	3.98	94.47	104.74	89.55	3.9	4.1	95.12	108.64	92.25

61	2.76	3.08	89.61	76.12	69.49	2.95	3.1	95.16	81.37	69.94	3.56	3.97	89.67	129.37	110.07	3.7	4	92.50	134.46	110.91
62	2.3	2.72	84.56	84.88	79.88	2.4	2.7	88.89	88.57	79.29	3.77	4.43	85.10	131.13	120.47	4.1	4.5	91.11	142.60	122.38
63	2.43	3.45	70.43	91.97	99.80	2.5	3.5	71.43	94.62	101.24	3.54	4.2	84.29	130.64	123.34	3.8	4.3	88.37	140.24	126.27
64	3.12	4.05	77.04	115.54	115.32	3.3	4.1	80.49	122.21	116.74	2.55	4.05	62.96	94.11	118.93	2.8	3.7	75.68	103.33	108.65
65	2.67	3.66	72.95	79.54	86.64	2.81	3.71	75.74	83.71	87.83	3.68	4.41	83.45	135.81	129.50	3.95	4.4	89.77	145.78	129.21
66	2.55	3.15	80.95	75.16	74.79	2.76	3.2	86.25	81.35	75.97	2.88	4.18	68.90	112.73	123.87	3	4.2	71.43	117.43	124.47
67	2.55	3.18	80.19	78.53	78.05	2.67	3.2	83.44	82.23	78.54	2.87	3.41	84.16	103.46	92.82	2.91	3.48	83.62	104.90	94.73
68	2.67	3.36	79.46	104.51	99.57	2.81	3.41	82.40	109.99	101.05	3.65	4.23	86.29	134.70	124.22	3.7	4.3	86.05	136.55	126.27
69	3.12	3.6	86.67	104.29	95.06	3.21	3.58	89.66	107.30	94.53	3.89	4.2	92.62	131.61	110.55	4	4.3	93.02	135.33	113.18
70	3.1	4.26	72.77	114.41	125.10	3.08	4.31	71.46	113.67	126.57	4.13	4.84	85.33	152.42	142.13	4.2	4.9	85.71	155.00	143.89
71	2.54	3.07	82.74	80.96	78.22	2.6	3.1	83.87	82.87	78.98	3.65	4.18	87.32	107.59	99.24	3.7	4.2	88.10	109.06	99.71
72	3.45	4.62	74.68	100.17	107.27	3.6	4.7	76.60	104.53	109.13	3.67	4.42	83.03	135.44	129.80	3.7	4.5	82.22	136.55	132.15
73	3.32	3.9	85.13	142.70	133.16	3.47	4.01	86.53	149.14	136.92	3.56	4.67	76.23	124.41	124.32	3.8	4.9	77.55	132.80	130.45
74	3.12	3.48	89.66	115.14	102.19	3.19	3.52	90.63	117.73	103.37	3.81	4.27	89.23	107.01	96.68	3.9	4.4	88.64	109.53	99.62
75	3.14	3.77	83.29	111.74	102.96	3.27	3.81	85.83	116.37	104.05	3.98	4.65	85.59	146.88	136.55	4	4.7	85.11	147.62	138.02
76	3.65	3.98	91.71	125.68	107.43	3.7	4	92.50	127.40	107.97	3.67	4.55	80.66	122.32	115.67	3.8	4.6	82.61	126.65	116.94
77	2.98	3.97	75.06	92.41	99.07	3.31	4.02	82.34	102.65	100.32	3.02	4.3	70.23	103.43	112.82	3.2	4.5	71.11	109.60	118.07
78	2.78	2.55	109.02	101.02	70.70	3	2.6	115.38	109.02	72.09	3.87	4.6	84.13	133.97	127.85	4.1	4.8	85.42	141.93	133.41
79	3.25	3.96	82.07	99.41	95.62	3.4	4	85.00	104.00	96.58	3.9	4.85	80.41	107.97	107.51	4.2	4.9	85.71	116.28	108.61
80	3.27	3.97	82.37	120.68	116.58	3.31	4.01	82.54	122.16	117.76	3.77	3.96	95.20	139.13	116.29	3.89	4.01	97.01	143.56	117.76
81	3.45	4.1	84.15	102.10	95.54	3.5	4.2	83.33	103.58	97.87	2.78	3.84	72.40	102.60	112.77	2.81	3.89	72.24	103.70	114.23
82	3.35	3.91	85.68	123.63	114.82	3.41	3.96	86.11	125.85	116.29	3.67	4.65	78.92	108.18	110.40	4	4.7	85.11	117.90	111.58
83	3.55	4.15	85.54	133.33	124.32	3.6	4.2	85.71	135.21	125.82	3.44	4.25	80.94	110.19	111.31	3.5	4.3	81.40	112.12	112.62
84	3.25	4.21	77.20	101.70	105.78	3.3	4.3	76.74	103.27	108.04	3.78	4.7	80.43	115.14	115.70	3.81	4.75	80.21	116.06	116.93
85	3.7	4.26	86.85	119.30	108.21	3.9	4.3	90.70	125.75	109.22	3.44	4.16	82.69	126.95	122.16	3.48	4.21	82.66	128.43	123.63
86											3.87	4.45	86.97	118.62	106.43	4	4.5	88.89	122.61	107.63
87											2.67	3.4	78.53	98.54	99.84	2.88	3.49	82.52	106.29	102.49
88											2.91	3.39	85.84	103.56	92.58	2.98	3.44	86.63	106.05	93.95
89											3.57	4.55	78.46	131.75	133.61	3.75	4.6	81.52	138.39	135.08
90											3.89	4.7	82.77	125.43	119.38	4.1	4.9	83.67	132.20	124.46
91											3.12	3.15	99.05	97.85	78.37	3.21	3.21	100.00	100.67	79.86
92											2.87	3.66	78.42	105.92	107.48	2.98	3.71	80.32	109.98	108.95
93											2.76	3.6	76.67	96.00	97.90	2.98	3.71	80.32	103.65	100.89
94											4.1	4.61	88.94	151.31	135.38	4.2	4.7	89.36	155.00	138.02
95											3.65	4.3	84.88	134.70	126.27	3.8	4.3	88.37	140.24	126.27
96											3.11	4.45	69.89	116.72	126.27	3.2	4.5	71.11	120.10	127.69
97											3.11	3.73	83.38	95.78	91.55	3.21	3.78	84.92	98.86	92.77
98											2.76	3.56	77.53	85.00	87.37	2.9	3.6	80.56	89.31	88.35
99											3.71	4.25	87.29	136.92	124.81	3.8	4.3	88.37	140.24	126.27
100											2.54	3.2	79.38	103.89	99.23	2.6	3.3	78.79	106.34	102.33
101											3.21	4.3	74.65	118.47	126.27	3.4	4.4	77.27	125.48	129.21
102											2.82	2.96	95.27	106.73	85.62	2.97	3.01	98.67	112.41	87.07
103											3.65	4.15	87.95	134.70	121.87	3.8	4.2	90.48	140.24	123.34
104											3.12	4.35	71.72	88.18	100.01	3.2	4.4	72.73	90.44	101.16
105											3.13	3.85	81.30	84.96	85.80	3.4	3.9	87.18	92.29	86.91
106											3.3	4.55	72.53	113.89	121.52	3.4	4.6	73.91	117.35	122.86
107											3.45	4.35	79.31	127.32	127.74	3.7	4.4	84.09	136.55	129.21
108											3.24	4.25	76.24	122.63	122.94	3.4	4.3	79.07	128.69	124.39
109											2.76	4.15	66.51	102.47	116.85	2.9	4	72.50	107.67	112.62
110											3.2	3.5	91.43	87.72	77.79	3.3	3.55	92.96	90.46	78.90
111											3.7	4.07	90.91	119.57	102.35	3.8	4.1	92.68	122.80	103.11
112											3.18	4	79.50	117.36	117.46	3.3	4.1	80.49	121.79	120.40
113											3.77	4.25	88.71	105.68	97.09	3.9	4.3	90.70	109.32	98.24
114											3.72	4.32	86.11	132.38	117.98	3.7	4.4	84.09	131.67	120.16
115											3.44	4.01	85.79	109.65	102.17	3.6	4.1	87.80	114.75	104.46
116											3.67	4.25	86.35	122.04	109.14	3.8	4.3	88.37	126.37	110.43
117											3.76	4.05	92.84	121.51	101.85	3.9	4.1	95.12	126.03	103.11
118											3.88	4.55	85.27	129.99	118.90	4	4.6	86.96	134.01	120.20
119											2.78	3.75	74.13	99.97	103.19	2.9	3.8	76.32	104.28	104.56
120											3.56	4.35	81.84	109.64	106.76	3.7	4.4	84.09	113.95	107.99
121											3.55	4.45	79.78	131.01	130.68	3.6	4.5	80.00	132.86	132.15
122											3	2.98	100.67	100.28	78.69	3.01	2.97	101.35	100.61	78.42
123											2.45	3.16	77.53	156.50	171.60	2.5	3.2	78.13	159.69	173.77
124											3.45	4.35	79.31	127.32	127.74	3.6	4.4	81.82	132.86	129.21
125											3.23	3.75	86.13	113.49	102.75	3.4	3.8	89.47	119.47	104.12
126											3.55	4.5	78.89	96.37	100.28	3.7	4.6	80.43	100.44	102.51
127											3.23	3.97	81.36	119.20	116.58	3.38	4.02	84.08	124.74	118.05
128											3.01	2.98	101.01	111.08	87.51	3.08	3.03	101.65	113.67	88.98
129											3.45	4.25	81.18	122.77	116.07	3.6	4.3	83.72	128.11	117.43
130											2.62	3.45	75.94	81.64	88.45	2.6	3.5	74.29	81.02	89.73
131											3.45	4.1	84.15	127.32	120.40	3.6	4.3	83.72	132.86	126.27
132											3.45	4.35	79.31	105.09	107.08	3.5	4.4	79.55	106.61	108.31
133											4.1	4.4	93.18	129.68	112.65	4.2	4.5	93.33	132.84	115.21
134											3.3	4.25	77.65	104.90	104.39	3.4	4.3	79.07	108.08	105.62

135											3.56	3.95	90.13	112.67	98.95	3.7	4	92.50	117.10	100.20
136											3.34	4.35	76.78	113.00	114.50	3.4	4.4	77.27	115.03	115.81
137											3.52	4.16	84.62	122.43	113.13	3.8	4.3	88.37	132.17	116.94
138											3.3	4.3	76.74	117.51	123.71	3.4	4.4	77.27	121.08	126.59
139											3.23	4.15	77.83	94.21	98.81	3.3	4.2	78.57	96.25	100.00
140											3.65	4.18	87.32	112.65	101.60	3.8	4.2	90.48	117.28	102.09
141											3.54	4.3	82.33	111.97	102.09	3.6	4.4	81.82	113.87	104.46
142											3.45	4.4	78.41	109.12	112.65	3.6	4.5	80.00	113.87	115.21
143											3.55	4.2	84.52	100.97	98.07	3.6	4.3	83.72	102.39	100.41
144											3.54	4.45	79.55	118.33	117.50	3.8	4.5	84.44	127.02	118.82
145											2.45	3.45	71.01	82.89	90.81	2.6	3.5	74.29	87.97	92.12
146											3.55	4.35	81.61	112.28	111.37	3.6	4.4	81.82	113.87	112.65
147											3.67	4.35	84.37	113.03	106.76	3.9	4.4	88.64	120.11	107.99
148											3.67	4.35	84.37	140.82	125.39	3.9	4.4	88.64	149.65	126.84
149											3.78	4.44	85.14	139.50	130.38	4.1	4.5	91.11	151.31	132.15
150											2.76	3.75	73.60	108.40	116.17	2.9	3.8	76.32	113.90	117.72
151											3.67	4.4	83.41	114.29	107.67	3.7	4.5	82.22	115.23	110.12
152											3.87	4.04	95.79	137.72	110.33	4	4.1	97.56	142.35	111.97
153											3.65	4.18	87.32	100.06	92.90	3.8	4.2	90.48	104.17	93.35
154											3.14	4.23	74.23	104.96	111.69	3.2	4.3	74.42	106.97	113.54
155											2.98	3.92	76.02	106.05	107.05	3.01	4.01	75.06	107.12	109.51
156											3.6	4.23	85.11	123.30	110.98	3.8	4.3	88.37	130.15	112.82
157											3.67	4.18	87.80	117.49	104.40	3.7	4.2	88.10	118.45	104.90
158											3.56	4.12	86.41	129.69	112.99	3.6	4.3	83.72	131.15	117.93
159											3.56	4.05	87.90	112.19	103.51	3.6	4.1	87.80	113.45	104.79
160											3.7	4.72	78.39	141.70	145.52	3.9	4.8	81.25	149.36	147.98
161											3.6	4.56	78.95	132.86	133.91	3.8	4.6	82.61	140.24	135.08
