# Supplementary material for: Association between chronic obstructive pulmonary disease (COPD) and occupational exposures: A hospital based quantitative cross-sectional study among the Bangladeshi population
Source: PLoS One. 2020 Sep 23;15(9):e0239602. doi: 10.1371/journal.pone.0239602 (PMC7510960; doi:10.1371/journal.pone.0239602)
Supplement: S2 Table — (PDF) [file pone.0239602.s002.pdf]

## S2 Table: Spirometry values of COPD participants according to self-reported occupational exposure.

| | Exposed | | | | | | | | | | Unexposed | | | | | | | | | |
|---|---|---|---|---|---|---|---|---|---|---|---|---|---|---|---|---|---|---|---|---|
| | Pre-BD | | | | | Post-BD | | | | | Pre-BD | | | | | Post-BD | | | | |
| No. | FEV1 | FVC | FEV1/FVC | %FEV1 predicted | %FVC predicted | FEV1 | FVC | FEV1/FVC | %FEV1 predicted | % FVC predicted | FEV1 | FVC | FEV1/FVC | %FEV1 Predicted | % FVC Predicted | FEV1 | FVC | FEV1/FVC | %FEV1 Predicted | % FVC predicted |
| 01 | 2.3 | 3.4 | 67.65 | 69.26 | 80.26 | 2.31 | 3.45 | 66.96 | 69.56 | 81.44 | 1.77 | 3.05 | 58.03 | 65.88 | 84.93 | 1.87 | 3.1 | 60.32 | 69.60 | 86.32 |
| 02 | 2.36 | 3.88 | 60.82 | 64.69 | 86.24 | 2.46 | 3.9 | 63.08 | 67.44 | 86.68 | 1.75 | 2.85 | 61.40 | 67.55 | 84.76 | 1.79 | 2.9 | 61.72 | 69.09 | 86.25 |
| 03 | 2.22 | 3.44 | 64.53 | 66.14 | 81.44 | 2.3 | 3.5 | 65.71 | 68.52 | 82.86 | 2.8 | 4.2 | 66.67 | 103.69 | 119.59 | 3 | 4.3 | 69.77 | 111.10 | 122.44 |
| 04 | 2.67 | 3.98 | 67.09 | 101.06 | 115.13 | 2.8 | 4.05 | 69.14 | 105.98 | 117.15 | 2.49 | 4 | 62.25 | 81.28 | 106.30 | 2.8 | 4.2 | 66.67 | 91.40 | 111.61 |
| 05 | 1.96 | 3.05 | 64.26 | 66.31 | 80.28 | 2.06 | 3.07 | 67.10 | 69.70 | 80.80 | 2.36 | 3.6 | 65.56 | 83.98 | 98.32 | 2.4 | 3.7 | 64.86 | 85.41 | 101.05 |
| 06 | 2.25 | 3.88 | 57.99 | 61.08 | 86.47 | 2.55 | 3.9 | 65.38 | 69.22 | 86.91 | 2.59 | 4.22 | 61.37 | 91.01 | 115.63 | 2.8 | 4.2 | 66.67 | 98.38 | 115.08 |
| 07 | 2.23 | 3.78 | 58.99 | 63.43 | 88.27 | 2.4 | 3.75 | 64.00 | 68.26 | 87.56 | 2.76 | 4.15 | 66.51 | 101.86 | 113.71 | 2.8 | 4.2 | 66.67 | 103.33 | 115.08 |
| 08 | 1.7 | 2.98 | 57.05 | 61.82 | 87.12 | 1.92 | 3 | 64.00 | 69.82 | 87.70 | 2.65 | 4.45 | 59.55 | 88.32 | 113.13 | 2.9 | 4.5 | 64.44 | 96.66 | 114.40 |
| 09 | 2.12 | 3.65 | 58.08 | 59.92 | 83.91 | 2.4 | 3.7 | 64.86 | 67.83 | 85.06 | 2.34 | 3.55 | 65.92 | 79.77 | 95.12 | 2.4 | 3.6 | 66.67 | 81.82 | 96.46 |
| 10 | 2.05 | 3.75 | 54.67 | 60.43 | 89.03 | 2.31 | 3.7 | 62.43 | 68.09 | 87.84 | 2.45 | 3.87 | 63.31 | 81.90 | 102.19 | 2.8 | 4.1 | 68.29 | 93.59 | 108.26 |
| 11 | 2.55 | 4.25 | 60.00 | 94.11 | 124.81 | 2.69 | 4.41 | 61.00 | 99.28 | 129.50 | 2.25 | 3.43 | 65.60 | 64.94 | 81.90 | 2.3 | 3.56 | 64.61 | 66.39 | 85.01 |
| 12 | 2.4 | 3.87 | 62.02 | 80.22 | 102.19 | 2.6 | 3.9 | 66.67 | 86.91 | 102.98 | 2.3 | 3.5 | 65.71 | 67.79 | 83.09 | 2.4 | 3.6 | 66.67 | 70.74 | 85.47 |
| 13 | 2.76 | 4.25 | 64.94 | 101.86 | 124.81 | 3 | 4.3 | 69.77 | 110.72 | 126.27 | 2.65 | 4.02 | 65.92 | 81.06 | 97.06 | 2.8 | 4.1 | 68.29 | 85.65 | 99.00 |
| 14 | 2.7 | 4.15 | 65.06 | 85.40 | 106.25 | 2.76 | 4 | 69.00 | 87.30 | 102.41 | 1.98 | 3.23 | 61.30 | 58.36 | 76.68 | 2.23 | 3.3 | 67.58 | 65.73 | 78.35 |
| 15 | 1.93 | 3.85 | 50.13 | 59.98 | 95.13 | 2 | 3.8 | 52.63 | 62.15 | 93.90 | 2.35 | 3.55 | 66.20 | 86.73 | 104.25 | 2.4 | 3.5 | 68.57 | 88.57 | 102.78 |
| 16 | 2.65 | 4.1 | 64.63 | 85.45 | 104.14 | 2.8 | 4.3 | 65.12 | 90.28 | 109.22 | 2.32 | 4.05 | 57.28 | 85.91 | 115.32 | 2.5 | 4.1 | 60.98 | 92.58 | 116.74 |
| 17 | 2.67 | 4.2 | 63.57 | 98.54 | 123.34 | 3 | 4.3 | 69.77 | 110.72 | 126.27 | 2.54 | 3.78 | 67.20 | 81.90 | 96.02 | 2.6 | 3.87 | 67.18 | 83.84 | 98.30 |
| 18 | 2.55 | 4.22 | 60.43 | 80.65 | 108.04 | 2.7 | 4.2 | 64.29 | 85.40 | 107.53 | 2.54 | 3.66 | 69.40 | 80.05 | 93.54 | 2.6 | 3.8 | 68.42 | 81.94 | 97.12 |
| 19 | 2.45 | 3.44 | 71.22 | 90.42 | 101.02 | 2.5 | 3.6 | 69.44 | 92.26 | 105.72 | 2.25 | 3.16 | 71.20 | 70.91 | 80.76 | 2.2 | 3.2 | 68.75 | 69.33 | 81.78 |
| 20 | 2.45 | 3.6 | 68.06 | 86.14 | 103.93 | 2.5 | 3.7 | 67.57 | 87.90 | 106.82 | 2.22 | 3.33 | 66.67 | 73.33 | 88.21 | 2.2 | 3.4 | 64.71 | 72.67 | 90.06 |
| 21 | 2.55 | 3.87 | 65.89 | 96.51 | 111.95 | 2.6 | 3.9 | 66.67 | 98.41 | 112.82 | 2.05 | 2.99 | 68.56 | 79.13 | 88.93 | 2.07 | 3.01 | 68.77 | 79.90 | 89.52 |
| 22 | 2.45 | 3.55 | 69.01 | 74.63 | 87.39 | 2.5 | 3.76 | 66.49 | 76.15 | 92.56 | 2.44 | 3.98 | 61.31 | 90.05 | 116.88 | 2.5 | 4 | 62.50 | 92.26 | 117.46 |
| 23 | 2.4 | 3.6 | 66.67 | 67.83 | 82.77 | 2.55 | 3.7 | 68.92 | 72.07 | 85.06 | 2.45 | 4.36 | 56.19 | 81.90 | 115.12 | 2.9 | 4.4 | 65.91 | 96.94 | 116.18 |
| 24 | 2.78 | 4.2 | 66.19 | 87.93 | 107.53 | 2.9 | 4.3 | 67.44 | 91.73 | 110.09 | | | | | | | | | | |
| 25 | 2.2 | 3.5 | 62.86 | 70.12 | 89.18 | 2.3 | 3.56 | 64.61 | 73.31 | 90.71 | | | | | | | | | | |
| 26 | 2.5 | 4.2 | 59.52 | 92.26 | 123.34 | 2.6 | 4.1 | 63.41 | 95.95 | 120.40 | | | | | | | | | | |
| 27 | 2.76 | 4.35 | 63.45 | 102.21 | 123.86 | 2.8 | 4.4 | 63.64 | 103.69 | 125.28 | | | | | | | | | | |
| 28 | 2.4 | 3.84 | 62.50 | 69.55 | 89.99 | 2.6 | 4 | 65.00 | 75.34 | 93.74 | | | | | | | | | | |
| 29 | 1.92 | 3.3 | 58.18 | 65.80 | 91.02 | 1.98 | 3.4 | 58.24 | 67.86 | 93.78 | | | | | | | | | | |
| 30 | 2.1 | 3.65 | 57.53 | 68.86 | 95.00 | 2.1 | 3.3 | 63.64 | 68.86 | 85.89 | | | | | | | | | | |
| 31 | 2.15 | 3.1 | 69.35 | 81.22 | 95.93 | 2.1 | 3.2 | 65.63 | 79.33 | 99.03 | | | | | | | | | | |
| 32 | 2.05 | 3.95 | 51.90 | 60.43 | 93.78 | 2.35 | 3.9 | 60.26 | 69.27 | 92.59 | | | | | | | | | | |
| 33 | 2.03 | 3.5 | 58.00 | 67.86 | 92.42 | 2.3 | 3.45 | 66.67 | 76.88 | 91.10 | | | | | | | | | | |
| 34 | 2.09 | 3.1 | 67.42 | 69.86 | 81.85 | 2.15 | 3.2 | 67.19 | 71.87 | 84.49 | | | | | | | | | | |
| 35 | 2.3 | 3.7 | 62.16 | 87.28 | 105.82 | 2.6 | 3.8 | 68.42 | 98.66 | 108.68 | | | | | | | | | | |
| 36 | 2.2 | 3.07 | 71.66 | 88.37 | 91.40 | 2.2 | 3.2 | 68.75 | 88.37 | 95.27 | | | | | | | | | | |
| 37 | 2.12 | 3.2 | 66.25 | 88.00 | 98.86 | 2.2 | 3.34 | 65.87 | 91.32 | 103.19 | | | | | | | | | | |
| 38 | 2.2 | 3.6 | 61.11 | 78.29 | 98.32 | 2.5 | 3.7 | 67.57 | 88.97 | 101.05 | | | | | | | | | | |
| 39 | 2.33 | 3.6 | 64.72 | 67.96 | 85.71 | 2.35 | 3.8 | 61.84 | 68.54 | 90.48 | | | | | | | | | | |
| 40 | 2.67 | 4.1 | 65.12 | 98.54 | 120.40 | 2.7 | 4 | 67.50 | 99.64 | 117.46 | | | | | | | | | | |
| 41 | 2.6 | 4 | 65.00 | 95.95 | 117.46 | 2.67 | 4 | 66.75 | 98.54 | 117.46 | | | | | | | | | | |
| 42 | 2.1 | 3.55 | 59.15 | 71.59 | 95.12 | 2.2 | 3.5 | 62.86 | 75.00 | 93.78 | | | | | | | | | | |
| 43 | 2.34 | 3.5 | 66.86 | 86.66 | 99.66 | 2.3 | 3.6 | 63.89 | 85.17 | 102.50 | | | | | | | | | | |
| 44 | 2.34 | 3.4 | 68.82 | 68.97 | 80.72 | 2.4 | 3.5 | 68.57 | 70.74 | 83.09 | | | | | | | | | | |
| 45 | 2.15 | 3.1 | 69.35 | 62.43 | 71.98 | 2.2 | 3.2 | 68.75 | 63.88 | 74.30 | | | | | | | | | | |
| 46 | 2.82 | 4 | 70.50 | 83.12 | 94.97 | 2.8 | 4.1 | 68.29 | 82.53 | 97.34 | | | | | | | | | | |
| 47 | 2.55 | 3.95 | 64.56 | 83.80 | 101.76 | 2.6 | 4 | 65.00 | 85.44 | 103.04 | | | | | | | | | | |
| 48 | 2.23 | 3.85 | 57.92 | 64.37 | 91.93 | 2.3 | 3.9 | 58.97 | 66.39 | 93.13 | | | | | | | | | | |
| 49 | 2.2 | 3.6 | 61.11 | 69.63 | 90.18 | 2.3 | 3.5 | 65.71 | 72.79 | 87.68 | | | | | | | | | | |
| 50 | 2.2 | 3.2 | 68.75 | 68.22 | 79.85 | 2.25 | 3.3 | 68.18 | 69.78 | 82.35 | | | | | | | | | | |
| 51 | 2.65 | 4.22 | 62.80 | 102.29 | 125.51 | 3 | 4.3 | 69.77 | 115.80 | 127.89 | | | | | | | | | | |
| 52 | 2.72 | 4.5 | 60.44 | 82.46 | 107.94 | 3.1 | 4.6 | 67.39 | 93.98 | 110.34 | | | | | | | | | | |
| 53 | 2.15 | 3.12 | 68.91 | 64.05 | 73.86 | 2.22 | 3.2 | 69.38 | 66.14 | 75.76 | | | | | | | | | | |
| 54 | 2.88 | 4.2 | 68.57 | 91.35 | 104.18 | 2.9 | 4.3 | 67.44 | 91.98 | 106.66 | | | | | | | | | | |
| 55 | 2.92 | 4.25 | 68.71 | 92.42 | 106.47 | 3 | 4.3 | 69.77 | 94.95 | 107.72 | | | | | | | | | | |
| 56 | 2.65 | 3.95 | 67.09 | 97.80 | 116.00 | 2.6 | 4 | 65.00 | 95.95 | 117.46 | | | | | | | | | | |
| 57 | 2.76 | 4.23 | 65.25 | 96.98 | 115.90 | 2.9 | 4.3 | 67.44 | 101.90 | 117.82 | | | | | | | | | | |
| 58 | 2.1 | 3.13 | 67.09 | 66.00 | 77.11 | 2.2 | 3.2 | 68.75 | 69.14 | 78.84 | | | | | | | | | | |
| 59 | 2.71 | 4.3 | 63.02 | 85.72 | 110.09 | 2.8 | 4.4 | 63.64 | 88.56 | 112.65 | | | | | | | | | | |

| | | | | | | | | | | | | | | | | | | | | |
|---|---|---|---|---|---|---|---|---|---|---|---|---|---|---|---|---|---|---|---|---|
| 60 | 2.76 | 4.45 | 62.02 | 84.07 | 109.54 | 2.8 | 4.5 | 62.22 | 85.29 | 110.77 | | | | | | | | | | |
| 61 | 2.45 | 3.7 | 66.22 | 79.00 | 93.98 | 2.5 | 3.8 | 65.79 | 80.61 | 96.52 | | | | | | | | | | |
| 62 | 2.43 | 3.5 | 69.43 | 89.68 | 102.78 | 2.5 | 3.7 | 67.57 | 92.26 | 108.65 | | | | | | | | | | |
| 63 | 2.67 | 4.05 | 65.93 | 84.45 | 103.69 | 2.7 | 4.1 | 65.85 | 85.40 | 104.97 | | | | | | | | | | |
| 64 | 2.45 | 4.25 | 57.65 | 90.42 | 124.81 | 2.7 | 4.3 | 62.79 | 99.64 | 126.27 | | | | | | | | | | |
| 65 | 1.96 | 3.12 | 62.82 | 53.63 | 69.96 | 1.9 | 3.2 | 59.38 | 51.99 | 71.75 | | | | | | | | | | |
| 66 | 2.1 | 3.18 | 66.04 | 67.71 | 80.77 | 2.2 | 3.2 | 68.75 | 70.94 | 81.28 | | | | | | | | | | |
| 67 | 2.45 | 3.42 | 71.64 | 71.00 | 80.15 | 2.4 | 3.5 | 68.57 | 69.55 | 82.02 | | | | | | | | | | |
| 68 | 2.32 | 3.66 | 63.39 | 67.23 | 85.77 | 2.4 | 3.7 | 64.86 | 69.55 | 86.71 | | | | | | | | | | |
| 69 | 2.43 | 3.6 | 67.50 | 72.88 | 86.60 | 2.3 | 3.7 | 62.16 | 68.98 | 89.01 | | | | | | | | | | |
| 70 | 2.23 | 3.44 | 64.83 | 65.86 | 80.91 | 2.3 | 3.6 | 63.89 | 67.93 | 84.67 | | | | | | | | | | |
| 71 | 2.45 | 3.88 | 63.14 | 90.42 | 113.94 | 2.5 | 3.9 | 64.10 | 92.26 | 114.53 | | | | | | | | | | |
| 72 | 2.1 | 3.1 | 67.74 | 67.86 | 77.96 | 2.1 | 3.2 | 65.63 | 67.86 | 80.47 | | | | | | | | | | |
| 73 | 2.14 | 3.1 | 69.03 | 69.00 | 78.74 | 2.2 | 3.2 | 68.75 | 70.94 | 81.28 | | | | | | | | | | |
| 74 | 2.25 | 3.2 | 70.31 | 83.04 | 93.97 | 2.3 | 3.4 | 67.65 | 84.88 | 99.84 | | | | | | | | | | |
| 75 | 2.05 | 2.87 | 71.43 | 87.20 | 90.20 | 2 | 3.01 | 66.45 | 85.08 | 94.60 | | | | | | | | | | |
| 76 | 2.32 | 3.44 | 67.44 | 85.62 | 101.02 | 2.4 | 3.5 | 68.57 | 88.57 | 102.78 | | | | | | | | | | |
| 77 | 2.24 | 3.3 | 67.88 | 68.23 | 81.23 | 2.2 | 3.4 | 64.71 | 67.01 | 83.69 | | | | | | | | | | |
| 78 | 2.31 | 3.65 | 63.29 | 74.48 | 92.71 | 2.6 | 3.8 | 68.42 | 83.84 | 96.52 | | | | | | | | | | |
| 79 | 2.1 | 3.1 | 67.74 | 77.50 | 91.03 | 2.2 | 3.2 | 68.75 | 81.19 | 93.97 | | | | | | | | | | |
| 80 | 2.25 | 3.4 | 66.18 | 64.24 | 77.95 | 2.3 | 3.5 | 65.71 | 65.67 | 80.24 | | | | | | | | | | |
| 81 | 1.98 | 2.86 | 69.23 | 66.18 | 75.52 | 1.95 | 2.9 | 67.24 | 65.18 | 76.57 | | | | | | | | | | |
| 82 | 2.15 | 3.1 | 69.35 | 79.35 | 91.03 | 2.2 | 3.2 | 68.75 | 81.19 | 93.97 | | | | | | | | | | |
| 83 | 2.43 | 3.7 | 65.68 | 76.86 | 94.73 | 2.6 | 3.8 | 68.42 | 82.24 | 97.29 | | | | | | | | | | |
| 84 | 1.95 | 3.06 | 63.73 | 65.82 | 81.39 | 2.2 | 3.2 | 68.75 | 74.26 | 85.11 | | | | | | | | | | |
| 85 | 2.45 | 3.45 | 71.01 | 86.09 | 94.53 | 2.5 | 3.6 | 69.44 | 87.84 | 98.64 | | | | | | | | | | |
| 86 | 2 | 3.2 | 62.50 | 73.81 | 93.97 | 2.25 | 3.3 | 68.18 | 83.04 | 96.91 | | | | | | | | | | |
| 87 | 2.2 | 3.35 | 65.67 | 77.60 | 94.52 | 2.3 | 3.3 | 69.70 | 81.12 | 93.11 | | | | | | | | | | |
| 88 | 2.23 | 3.35 | 66.57 | 84.40 | 96.91 | 2.2 | 3.3 | 66.67 | 83.27 | 95.46 | | | | | | | | | | |
| 89 | 2.2 | 3.12 | 70.51 | 81.19 | 91.62 | 2.2 | 3.2 | 68.75 | 81.19 | 93.97 | | | | | | | | | | |
| 90 | 2.1 | 3.47 | 60.52 | 69.52 | 90.96 | 2.1 | 3.5 | 60.00 | 69.52 | 91.75 | | | | | | | | | | |
| 91 | 2.2 | 3.23 | 68.11 | 79.36 | 92.61 | 2.3 | 3.4 | 67.65 | 82.97 | 97.48 | | | | | | | | | | |
| 92 | 2.15 | 3.12 | 68.91 | 79.35 | 91.62 | 2.2 | 3.2 | 68.75 | 81.19 | 93.97 | | | | | | | | | | |
| 93 | 2.3 | 3.23 | 71.21 | 84.88 | 94.85 | 2.3 | 3.4 | 67.65 | 84.88 | 99.84 | | | | | | | | | | |
| 94 | 2.66 | 4.12 | 64.56 | 101.27 | 122.97 | 2.6 | 4.1 | 63.41 | 98.99 | 122.38 | | | | | | | | | | |
| 95 | 2.12 | 3.96 | 53.54 | 78.24 | 116.29 | 2.03 | 4.01 | 50.62 | 74.92 | 117.76 | | | | | | | | | | |
| 96 | 2.2 | 4.22 | 52.13 | 81.19 | 123.92 | 2.5 | 4.3 | 58.14 | 92.26 | 126.27 | | | | | | | | | | |
| 97 | 2.45 | 4.35 | 56.32 | 81.90 | 114.86 | 2.6 | 4.5 | 57.78 | 86.91 | 118.82 | | | | | | | | | | |
| 98 | 2.43 | 4.25 | 57.18 | 91.97 | 122.94 | 2.5 | 4.3 | 58.14 | 94.62 | 124.39 | | | | | | | | | | |
| 99 | 2.05 | 2.8 | 73.21 | 81.45 | 87.48 | 2 | 2.9 | 68.97 | 79.46 | 90.61 | | | | | | | | | | |
| 100 | 2.34 | 4.12 | 56.80 | 71.28 | 101.42 | 2.5 | 4.2 | 59.52 | 76.15 | 103.39 | | | | | | | | | | |
| 101 | 2.1 | 3.45 | 60.87 | 77.77 | 98.23 | 2.4 | 3.6 | 66.67 | 88.88 | 102.50 | | | | | | | | | | |
| 102 | 2.54 | 4.44 | 57.21 | 94.61 | 130.38 | 2.6 | 4.5 | 57.78 | 96.84 | 132.15 | | | | | | | | | | |
| 103 | 2.65 | 4.18 | 63.40 | 100.89 | 124.77 | 2.6 | 4.2 | 61.90 | 98.99 | 125.36 | | | | | | | | | | |
| 104 | 2.4 | 3.5 | 68.57 | 73.96 | 90.01 | 2.45 | 3.6 | 68.06 | 75.50 | 92.58 | | | | | | | | | | |
