# Supplementary material for: Association between chronic obstructive pulmonary disease (COPD) and occupational exposures: A hospital based quantitative cross-sectional study among the Bangladeshi population
Source: PLoS One. 2020 Sep 23;15(9):e0239602. doi: 10.1371/journal.pone.0239602 (PMC7510960; doi:10.1371/journal.pone.0239602)
Supplement: Attachment — Submitted filename: Resonse to Reviewers.docx [file pone.0239602.s003.docx]

Dated: 14^th^ August, 2020

The Editor

PLOS ONE

Subject: Response to Revision of Manuscript titled “‘Association between chronic obstructive pulmonary disease (COPD) and occupational exposures: A hospital based quantitative cross-sectional study among the Bangladeshi population.”

Dear Respected Editor,

Many thanks for sending the reviewers’ comments on our paper. We really appreciate that you gave us responses in such a short time. We also heartedly thank the reviewers for their valuable comments on the manuscript. We have edited the manuscript according to their suggestions and submitted accordingly. We are grateful enough to receive the inputs they have given for our manuscript. We strongly believe that their inputs will help us improving the quality of our manuscript substantially.

Please find the detailed responses to reviewers’ comments in the following page.

Sincerely,

Ahmed Faisal Sumit

(Corresponding author)

On behalf of all authors

Manuscript Number: PONE-D-20-16525

**Response to Reviewer’s Comments**

| **Reviewer 1 comment** | **Response** |
| --- | --- |
| As all of the patients were being evaluated for respiratory problems, the authors need to be very cautious in how these results are presented and interpreted. For example- what diagnoses did the patients in the control group actually have? | Initially, all the control participants were examined by registered physician after complaining about respiratory problems. They all were confirmly diagnosed without COPD based on the spirometry results that was FEV1/FVC>0.70, and FEV1% ≥ 80 predicted.  Based on the suggestions by registered physician, some of the control participants were also undergoing other tests like chest x-ray, serum IgE test, blood eosinophil count, etc, to confirm the diagnosis of asthma, bronchitis, emphysema, restrictive problems and/or other respiratory diseases. We have excluded those control participants who had been diagnosed with asthma, chronic bronchitis, and/or emphysema by registered physician during the time of study or if they had past history of these diseases. This information has been added in the manuscript now.  We have not excluded control participants if they had been diagnosed with restrictive problems. Besides, control participants with doctor diagnosed allergic rhinitis, wheezing, mild/chronic cough, morning phlegm, dyspnoea were also included in our study. However, majority of the control participants were diagnosed as normal by registered physician.  In our study we have only assessed self-reported respiratory symptoms of the participants (“Table 1”) rather than registered physician diagnosed respiratory diseases since we found majority of the control participants were diagnosed as normal by registered physician. |
| Also- the authors should present the proportion of patients with restriction (normal ratio and low FEV1)- As I suspect there would be a high proportion of patients with this abnormality. | According to your suggestion, we have included the proportion of control participants with restriction (normal ratio, but FVC<80% predicted) according to GOLD criteria. |
| Was pre bronchodilator lung function assessed? This might be important and including an assessment of bronchodilator responsiveness would be helpful. | According to your suggestion, we have included pre bronchodilator response data in our manuscript.  Since we excluded all control participants who have shown positive bronchodilator response due to the suspect of asthma, bronchodilator response was shown only for the COPD participants. |
| **Reviewer 2 comment** |  |
| The text is well written apart from 2 minimal mistakes. | We have addressed short grammatical mistakes and corrected accordingly. |
