# Supplementary material for: Association between chronic obstructive pulmonary disease (COPD) and occupational exposures: A hospital based quantitative cross-sectional study among the Bangladeshi population
Source: PLoS One. 2020 Sep 23;15(9):e0239602. doi: 10.1371/journal.pone.0239602 (PMC7510960; doi:10.1371/journal.pone.0239602)
Supplement: Attachment — Submitted filename: Resonse to Reviewers.docx [file pone.0239602.s004.docx]

Dated: 09^th^ September, 2020

The Editor

PLOS ONE

Subject: Response to Revision of Manuscript titled “‘Association between chronic obstructive pulmonary disease (COPD) and occupational exposures: A hospital based quantitative cross-sectional study among the Bangladeshi population.”

Dear Respected Editor,

Many thanks for sending the reviewer comments on our paper. We really appreciate that you gave us responses in such a short time. We also heartedly thank the reviewers for their valuable comments on the manuscript. We have edited the manuscript according to their suggestions and submitted accordingly. We are grateful enough to receive the inputs they have given for our manuscript. We strongly believe that their inputs will help us improving the quality of our manuscript substantially.

Please find the detailed responses to reviewer comments in the following page.

Sincerely,

Ahmed Faisal Sumit

(Corresponding author)

On behalf of all authors

Manuscript Number: PONE-D-20-16525

**Response to Reviewer Comments**

| **Reviewer comment** | **Response** |
| --- | --- |
| The association between occupational exposure and bronchodilator response among the COPD participants were also assessed in our study. Here, similar to earlier study [34], our study showed that 12-20% of COPD participants exhibited positive bronchodilator response, although the association was not significant when compared to occupational exposure. Our finding was in contrast with previous study showing the significant association between %FEV1 reversibility with occupational exposure among the COPD participants [23]. **However, such assessment was performed only based on the increase of average %FEV1 predicted values among the exposed group rather than on positive bronchodilator response."**  **The last sentence is unclear to me: do you refer to your study or to the previous one?** | The last sentence refers to previous study and we have added this in our manuscript as follow:  “However, such assessment in the previous study [23] was performed only based on the increase of average %FEV1 predicted values among the exposed group rather than on positive bronchodilator response.” |
